# *ERF49* Gene Negatively Regulates Plant Resistance to *Verticillium* Wilt Through Modulation of Genes Involved in Lignin Biosynthesis

**DOI:** 10.3390/ijms27083447

**Published:** 2026-04-12

**Authors:** Mingrui Li, Hang Ruan, Qi Mi, Baocheng Li, Wanyu Sha, Zhiquan Liu, Yajun Liang, Junduo Wang, Juyun Zheng, Zhaolong Gong, Zhonghua Zhou, Zhi Liu, Sujun Jiang, Shengwei Zhu, Wenyan Fan

**Affiliations:** 1 Agricultural College, Heilongjiang Bayi Agricultural University, Daqing 163319, China; 15046162090@163.com (M.L.); 13093390736@163.com (H.R.); 13234503131@163.com (Q.M.); 15765904550@163.com (B.L.); 18724666056@163.com (W.S.); jsjfwy@sohu.com (S.J.); 2 Key Laboratory of Plant Molecular Physiology, Institute of Botany, Chinese Academy of Sciences, Beijing 100093, China; zhqliu@ibcas.ac.cn; 3 Xinjiang Cotton Technology Innovation Center, Xinjiang Key Laboratory of Cotton Genetic Improvement and Intelligent Production, National Cotton Engineering Technology Research Center, Cotton Research Institute of Xinjiang Uyghur Autonomous Region Academy of Agricultural Sciences, Wulumuqi 830091, China; 13999966149@163.com (Y.L.); 13579975299@163.com (J.W.); zjypp8866@126.com (J.Z.); g15981775091@163.com (Z.G.); 4 College of Bioscience and Biotechnology, Hunan Agricultural University, Changsha 410128, China; zhouzhonghua1976@hotmail.com (Z.Z.); zhiliu@hunau.edu.cn (Z.L.)

**Keywords:** *ERF49* gene, *Verticillium* wilt, VIGS, disease resistance

## Abstract

Cotton *Verticillium* wilt seriously threatens global cotton production, necessitating the development of resistant cultivars through molecular breeding. Members of the ethylene response factor (ERF) family function as pivotal transcriptional regulators of the ethylene signaling pathway, orchestrating plant defensive responses against pathogen invasion. Here, through comprehensive phenotypic and transcriptional analyses of lignin biosynthesis genes in *AtERF49*-overexpressing lines, loss-of-function mutants, dominant repressor plants, and *GhERF49*-silenced cotton plants (TRV-VIGS), we demonstrate that *AtERF49* functions as a negative regulator of *Verticillium* wilt resistance. Overexpression of *AtERF49* significantly compromised defense responses in *Arabidopsis thaliana*, whereas *GhERF49* silencing enhanced cotton resistance to *Verticillium* wilt. Transcription analysis showed that *ERF49*-mediated susceptibility correlates with suppression of lignin biosynthesis-related genes following pathogen challenge, suggesting that *ERF49* interferes with inducible cell wall fortification. These findings elucidate a previously unrecognized negative regulatory node linking ethylene signaling to lignin-mediated disease resistance, providing promising biotechnological targets for engineering durable *Verticillium* wilt resistance in cotton and related crops.

## 1. Introduction

Cotton *Verticillium* wilt is a devastating vascular disease caused by *Verticillium dahliae* which severely compromises global cotton yield and fiber quality [1]. Conventional resistance breeding has been hampered by limited genetic diversity for resistance in upland cotton (*Gossypium hirsutum*) germplasm resources. Recent advances in molecular biology techniques have provided powerful strategies to elucidate plant disease resistance mechanisms [2]. Plants resist pathogen infection through multilayered immune responses. As central integrators of the ethylene signaling pathway, ethylene response factors (ERFs) play a pivotal role in regulating resistance gene expression, reactive oxygen species (ROS) burst, and stomatal immunity [3]. During plant–pathogen interactions, ERF genes participate in diverse regulatory pathways. Notably, ERF genes function as direct targets of the mitogen-activated protein kinase (MAPK) signaling cascade. Upon perception of plant pathogen attack, activated MAPKs phosphorylate ERF proteins, thereby modulating their activity and subcellular localization to regulate the expression of downstream defense genes [4,5]. In *Arabidopsis*, the MPK3/MPK6 phosphorylate *ERF72* directly activates the transcription of phytoalexin biosynthetic genes (*PAD3* and *CYP71A13*), and indirectly regulates the expression of *PAD3* and *CYP71A13* via the induction of the transcription factor *WRKY33*, thereby enhancing resistance against *Botrytis cinerea* [6].

Additionally, ERF genes serve as critical integrators of plant hormone signaling networks, and coordinate multilayered defense responses. Canonical plant defense hormones such as jasmonic acid (JA), salicylic acid (SA) and ethylene (ET) are central regulators of plant immune response. They activate or repress the expression of a series of defense genes through their respective signal transduction pathways [7,8]. The expression of ERF genes is dynamically modulated by these hormones, and individual ERF members serve as pivotal nodes in hormonal crosstalk [9,10]. For instance, while both *AtERF1* and *ORA59* operate at the convergence of ethylene and JA, signaling downstream pathways to orchestrate resistance against necrotrophic fungi, *ORA59* uniquely functions as a key regulatory node in the antagonistic interaction between SA and JA [11,12]. Similarly, in broccoli (*Brassica oleracea* var. *italica*), infection with the black rot pathogen *Xanthomonas campestris* pv. *Campestris* triggers SA-dependent signaling, which strongly induces the expression of AP2/ERF transcription factors; these factors subsequently regulate defense-related genes to initiate the defense response [13].

These findings establish a theoretical basis for molecular design breeding of *Verticillium* wilt resistance via ERF gene manipulation. However, critical knowledge gaps remain regarding the allelic diversity of ERF genes across diverse cotton germplasms, and their interaction dynamics with pathogen effector proteins. Leveraging our prior cloning of *GhERF49*, the cotton homolog of *Arabidopsis thaliana AtERF49*, we employed an integrated approach combining bioinformatics, molecular biology, and biochemistry to elucidate the functional role of *ERF49* in *Verticillium* wilt resistance. Through overexpression and loss-of-function (VIGS-mediated silencing) analyses, we demonstrated that *ERF49* functions as a negative regulator of plant immunity, with its suppression enhancing resistance against *Verticillium* wilt. These discoveries not only advance our understanding of integrating ethylene signaling into defense networks, but also provide immediately applicable genetic targets for developing resilient cotton cultivars through precision breeding.

## 2. Results

### 2.1. AtERF49 Increases Plant Susceptibility to Verticillium dahliae

**Figure 1:**
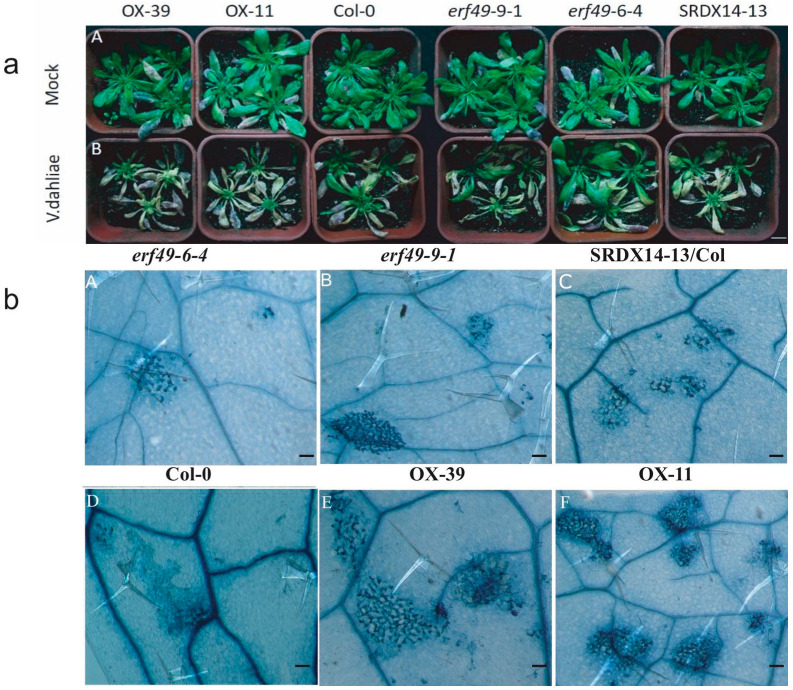
Overexpression of *AtERF49* increased the susceptibility of Arabidopsis to *Verticillium dahliae*. (**a**) (**A**,**B**) Disease symptoms of erf49-6-4, erf49-9-1, SRDX14-13/Col, Col-0, OX-39 and OX-11 plants at 14 days post-inoculation (dpi) with Czapek–Dox medium (Mock) or *Verticillium dahliae* (*V. dahliae*). Scale bar = 4 cm. (**b**) (**A**–**F**) Trypan blue staining showing cell death in leaves of indicated Arabidopsis genotypes. Scale bar = 100 µm.

Two-week-old *Arabidopsis thaliana* seedlings were inoculated with *Verticillium dahliae*, and the plant phenotype was observed for 14 days post-inoculation (dpi). The results showed that *AtERF49*-overexpression plants exhibited markedly enhanced susceptibility to *V. dahliae*, with severe wilting symptoms compared to wild-type Col-0 (Figure 1a). Conversely, *erf49* and ERF49-SRDX/Col mutants displayed milder wilting symptoms, indicating increased resistance to *V. dahlia* (Figure 1a). To quantify *V. dahliae*-induced cell death, *Arabidopsis* leaves were stained with trepan. Consistent with the results from the aforementioned phenotypic experiments, severe cell death was observed in AtERF49-OX plants after inoculation with *V. dahliae*, while both *erf49* mutants and SRDX-ERF49 plants showed mild cell death (Figure 1b). These results demonstrate that *AtERF49* negatively regulates the defense response of Arabidopsis to *V. dahliae*, as a susceptibility factor in the plant pathosystem.

### 2.2. Silencing GhERF49 Enhances Resistance to Verticillium Wilt in Cotton

**Figure 2:**
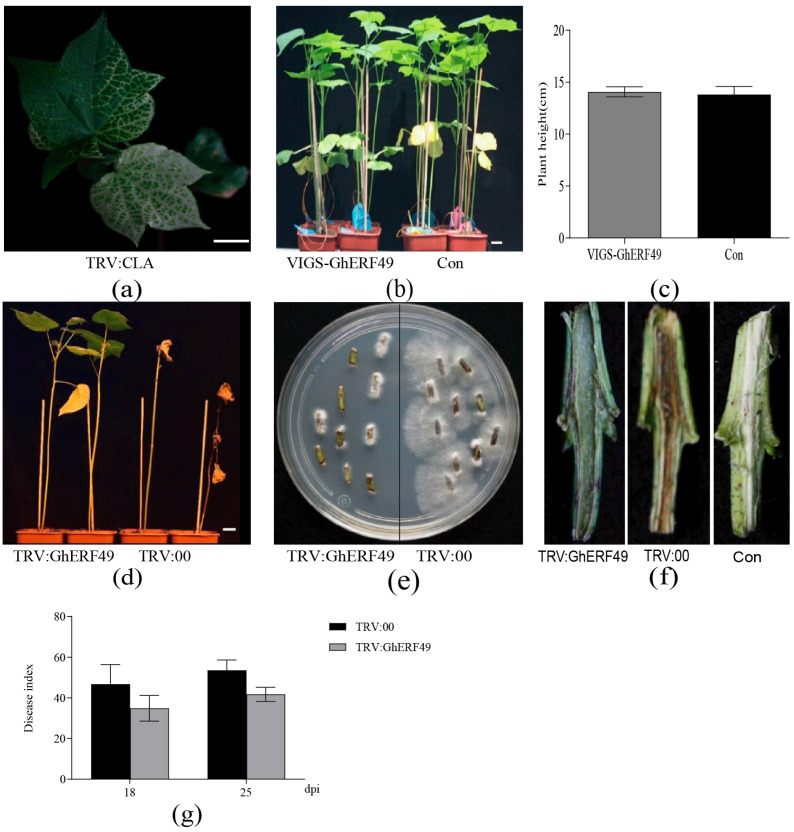
Silencing *GhERF49* enhances cotton resistance to Verticillium wilt. (**a**) Phenotype of VIGS-CLA plant at 14 days post-inoculation (dpi) with *Verticillium dahliae*. (**b**,**c**) Disease symptoms (**b**) and plant height (**c**) of VIGS -silenced plants at 2 weeks post-treatment. Con is control; VIGS-*GhERF49* is the silencing experimental group. (**d**) Phenotypic analysis of *GhERF49*-silenced plants compared to control at 35 dpi. Scale bar = 1 cm. (**e**) Recovery culture of the pathogen from stems. (**f**) Longitudinal sections of stem cuttings from TRV:00 and TRV:*GhERF49*, showing disease symptoms. (**g**) Disease indices of TRV:*GhERF49* and TRV:00 plants at 18 and 25 dpi. The experiments above were repeated 3 times. The error bar is the standard error.

The *CLA* gene in cotton is related to the synthesis of chlorophyll, and its suppression causes leaf albinism. To validate the efficiency of the VIGS system, *Agrobacterium tumefaciens* GV3101 solution containing pTRV2-GhCLA and pTRV1 vectors was mixed and infiltrated into cotyledons of 2-week-old cotton (TM-1) seedlings of uniform size. The newly emerging leaves showed an albino phenotype (Figure 2a). This result indicated that the VIGS system was successful and effectively silenced the expression of cotton *CLA* genes. Subsequently, identical procedures were applied to silence *GhERF49*. The cotton plants of VIGS-*GhERF49* and control were sampled separately, and target gene expression was detected by qRT-PCR. Plant height was measured at the two-true-leaf stage. As can be seen in Figure 2c, there was no significant difference in plant height between VIGS-*GhERF49* plants and control plants (TRV:00).

In order to explore the role of *GhERF49* in cotton resistance to *Verticillium* wilt, the expression levels of *GhERF49* were first detected in both VIGS-*GhERF49* plants (TRV:*GhERF49*) and control plants (TRV:00). Subsequently, VIGS-*GhERF49* plants and control plants were quantitatively irrigated with solution of *V. dahliae* LX2-1 cultured for 1 week. The results showed that the symptoms of *Verticillium* wilt in TRV:*GhERF49* plants were significantly less severe than those in TRV:00 plants (Figure 2d). Statistical disease indices showed lower values in TRV:*GhERF49* plants compared to control plants (TRV:00) (Figure 2g). Specifically, the disease indices of TRV:*GhERF49* plants were 34.9% and 41.78% at 18 and 27 dpi, respectively, with *V. dahliae*, while disease indices of TRV:00 plants reached 46.78% and 53.57% at the same time points. Fungal recovery culture showed that the amount of *V. dahliae* in stems of TRV:*GhERF49* plants was significantly lower than in TRV:00 plants (Figure 2e). Stem cutting assays showed that the vascular bundles of the stems of TRV:00 plants developed yellowish-brown discoloration, showing acute symptoms of Verticillium wilt (Figure 2f). The above results indicate that suppressing the expression of *GhERF49* enhances cotton resistance to *Verticillium* wilt.

### 2.3. GhERF49 Negatively Regulates Lignin-Synthesis-Related Genes

**Figure 3:**
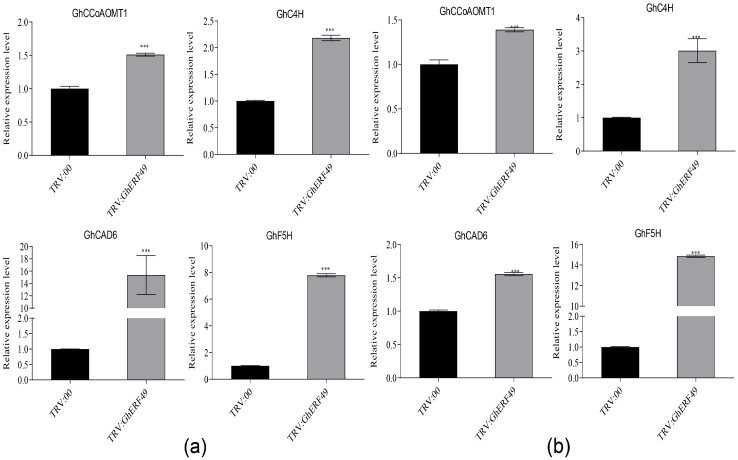
Expression analysis of lignin-related genes after silencing *GhERF49.* (**a**,**b**) Expression of pathway-related genes related to lignin synthesis in TRV:*GhERF49* and TRV:00 plants before and after Verticillium wilt treatment. *F5H1*—ferulate 5-hydroxylase 1; C4H—cinnamate 4-hydroxylase; *CAD6*—cinnamy1 alcohol dehydrogenase 6; *CCoAOMT1*—caffeoy1 CoA 3-O-methyltransferase 1. The asterisk indicates a statistically significant difference, *** indicates *p* < 0.001. The experiments above were repeated 3 times. The error bar is the standard error.

Lignin, a phenylpropanoid-derived polymer produced by oxidative polymerization of the three main hydroxycinnamyl alcohols, plays an essential role in plant defense against adverse environmental stresses [14]. To investigate whether *GhERF49* modulates lignin biosynthesis during *Verticillium dahlia* infection, we analyzed the transcription levels of key lignin biosynthesis genes in cotton. The results showed that transcription levels of four lignin formation genes (*GhCAD6*, *GhC4H1*, *GhF5H1* and *GhCCoAOMT1*) in TRV:*GhERF49* plants were significantly higher than those in TRV:00 plants before and after inoculation with *Verticillium dahliae* LX2-1. Before inoculation, expression levels of *GhCCoAOMT1*, *GhC4H1*, *GhCAD6* and *GhF5H1* in TRV:*GhERF49* plants were, respectively, 1.5, 2.2, 15.4, and 7.8 times higher than those in TRV:00 plants. Notably, *GhCAD6* exhibited the strongest induction (15.4-fold) (Figure 3a). After inoculation, expression of the four genes increased by 1.4-, 3.0-, 1.55- and 14.8-fold, respectively, with *GhF5H1* showing the most dramatic upregulation (14.8-fold) (Figure 3b). Taken together with the reduced disease symptoms in TRV:*GhERF49* plants (Figure 2d,f), these findings indicated that the improved resistance conferred by *GhERF49* silencing is mediated through the upregulation of lignin biosynthetic genes. Thus *GhERF49* functions as a negative regulator of lignin-mediated defense.

### 2.4. AtERF49 Negatively Regulates Expression of Lignin-Synthesis-Related Genes

**Figure 4:**
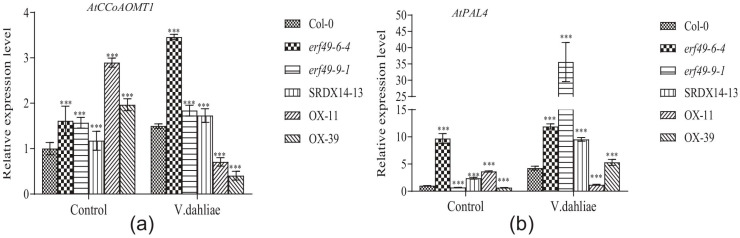
Expression analysis of lignin-related gene expression of *Arabidopsis.* Relative expression analysis of *AtCCoAOMT1* (**a**) and *AtPAL4* (**b**) genes. *AtUBC30* was used as reference gene. The asterisk indicates a statistically significant difference, *** indicates *p* < 0.001. The experiments above were repeated 3 times. The error bar is the standard error.

Based on the findings presented in Section 2.2 and Section 2.3, which showed that *GhERF49* silencing enhances cotton resistance to Verticillium wilt through depression of lignin biosynthesis, we hypothesized that its homolog in *Arabidopsis*, *AtERF49*, might function similarly. To validate this, the expression of lignin biosynthetic genes *AtPAL4* and *AtCCoAOMT1* was analyzed by qRT-PCR in *AtERF49* transgenic lines. As shown in Figure 4, the transcription levels of both genes differed significantly between transgenic *Arabidopsis* plants and wild-type Col-0 plants before *Verticillium dahlia* inoculation. After inoculation, *AtPAL4* and *AtCCoAOMT1* expression was markedly induced in *erf49* mutant and *ERF49* dominant repressor plants (*ERF49*-SRDX), with expression increases of 2- to 9-fold and 1- to 3-fold, respectively. Conversely, their expression was significantly repressed (50–73%) in *AtERF49*-OX plants. Combined with disease phenotypes (Figure 1a), overexpression of *AtERF49* increased the susceptibility of plants to Verticillium wilt, while inhibition of *ERF49* enhanced the resistance of *ERF49*-SRDX plants and erf49 mutants to Verticillium wilt. These findings from cotton and *Arabidopsis* established *ERF49* as a negative regulator of lignin biosynthesis-mediated defense against Verticillium wilt across dicot plants.

## 3. Discussion

This study identifies *ERF49* as a novel negative regulator of plant resistance to *Verticillium* wilt, potentially modulating susceptibility through transcriptional through of lignin biosynthesis. In the Arabidopsis model, *AtERF49* overexpression significantly suppressed the expression of lignin synthesis genes *AtPAL4* and *AtCCoAOMT1* (Figure 4) and enhance cell death (Figure 1b), resulting in enhanced susceptibility to *Verticillium* wilt.

Further supporting this conclusion, we found that this regulatory mechanism is also conserved in cotton, where silencing *GhERF49* in cotton significantly upregulated lignin biosynthetic genes (e.g., *GhCAD6* and *GhF5H1* (Figure 3) and reduced the disease severity by 34.9% (Figure 2g). These findings align with recent research indicating that lignin deposition resists vascular bundle diseases by enhancing cell wall barriers and reactive oxygen species (ROS) accumulation [15,16], while ERF transcription factors downstream of ethylene signals often function as immune balance switches [17]. It is worth noting that the function of *ERF49* is opposite to that of typical disease-resistant ERFs (e.g., *NbERF5*) [18], suggesting that there is a functional differentiation of the gene family.

The negative regulation mechanism of *ERF49* may involve hormone signal crosstalk and epigenetic modification. Studies have shown that jasmonic acid (JA) can activate lignin biosynthesis genes [19], and ethylene signaling inhibits JA pathway effectors via *ERF49*, resembling the antagonism of the *VaERF16*-JA module in grape vine [7]. Furthermore, histone *H3K27ac* modification was confirmed to relieve the inhibition of target genes by *ERF49* [20], which may explain why the expression of lignin genes in *GhERF49*-silenced plants was increased by 14.8-fold after *Verticillium dahliae* infection (Figure 3b).

The applicability of VIGS technology throughout the whole growth period of cotton (e.g., stem injection and fruit branch base injection) provides technical support for rapid screening in the field [21,22], while the functional conservation of *AtERF49* in *Arabidopsis thaliana* suggests that this mechanism may be conserved in dicots such as tomato, and soybean. It is noteworthy that Wang et al. [2] used host-induced gene silencing (HIGS) technology to target pathogen genes and enhance cotton disease resistance, providing a collaborative innovation path with the host gene editing strategy of this study. Combined with host-induced gene silencing (HIGS) targeting the pathogen *VdThit* gene [2], or co-expression with the disease-resistant positive regulator *GhWRKY75* [1], this strategy is expected to achieve dual-pathway enhancement of disease resistance. While our data demonstrate that *GhERF49* silencing leads to significant upregulation of key lignin biosynthesis genes (e.g., *GhCCoAOMT1*, *GhCAD6*) and enhanced disease resistance, the precise molecular mechanism warrants further investigation. Based on the present study, we cannot conclusively determine whether GhERF49 protein directly binds to the promoters of these lignin genes to repress their transcription, or whether it acts indirectly through intermediary regulators. The observed regulatory relationship may involve complex hormone signaling crosstalk (e.g., ethylene-JA antagonism) or epigenetic modifications, as suggested by prior studies in other systems. Future work employing chromatin immunoprecipitation sequencing (ChIP-seq), electrophoretic mobility shift assays (EMSA), or detailed promoter analysis will be essential to clarify whether these lignin genes are direct targets of *GhERF49*. Nevertheless, our findings firmly establish *GhERF49* as a critical negative regulator of lignin deposition and Verticillium wilt resistance, providing a valuable target for molecular design of resistant crop varieties [23].

This study reveals that the *ERF49* gene functions as a negative regulator of plant resistance to *Verticillium* wilt. In both *Arabidopsis* and cotton, loss-of-function or silencing of this gene significantly enhanced plant resistance, whereas its overexpression increased susceptibility.

The molecular mechanism involves *ERF49* negatively regulating the expression of several key lignin biosynthesis genes, such as *GhCAD6* and *GhC4H1*, thereby suppressing pathogen-induced lignification of the cell wall, a crucial defense response.

In summary, *ERF49* acts as a critical negative regulatory node linking ethylene signaling to lignin-mediated disease resistance. This finding provides a novel potential target for molecular breeding of disease-resistant crops. Whether *ERF49* directly regulates these downstream genes requires further investigation.

## 4. Materials and Methods

### 4.1. Plant Materials

The *Arabidopsis thaliana* material used in this experiment was ecotype Col-0. Plants were cultured at 22 °C under a 16 h light/8 h dark photoperiod.

The cotton materials used in the experiment was the upland cotton (*Gossypium hirsutum*) standard line TM-1, which was provided by the Key Laboratory of Plant Molecular Physiology, Chinese Academy of Sciences, Institute of Botany, Chinese Academy of Sciences. Plants were grown in a greenhouse with a day/night temperature cycle of 30 °C/22 °C, and a light cycle of 12 h light/12 h dark.

### 4.2. Strains and Carriers

The vectors used in cotton VIGS were pTRV1 and pTRV2. The *Agrobacterium tumefaciens* strain GV3101, used for transformation, was provided by the Key Laboratory of Plant Molecular Physiology, Institute of Botany, Chinese Academy of Sciences. *Verticillium dahliae* LX2-1 was provided by Hebei Agricultural University.

### 4.3. VIGS-Technology-Infected Cotton

*Agrobacterium* GV3101 carrying pTRV1/pTRV2-*GhERF49* was inoculated into LB liquid medium containing kanamycin (50 mg/L) and rifampicin (25 mg/L), then cultured at 28 °C and 200 rpm until OD_600_ = 0.5. After centrifugation at 5000 rpm for 15 min at 4 °C, the bacterial suspension was washed twice with sterile water to remove antibiotic residues, then re-suspended in the infection solution (10 mM MgCl2, 10 mM MES, 200 μM acetosyringone) and placed in the dark at room temperature for 4 h to induce infection. The pTRV1 was mixed with the target vector (such as pTRV2-*GhERF49*) at a ratio of 1:1, and the bacterial suspension was injected into the leaves after the backs of the cotton cotyledons were scratched with a needle until the leaves were completely penetrated. This protocol is suitable for 2-week-old seedlings (cotyledon expansion period), and can also be extended to 4-day-old stem injection or flowering fruit branch base injection to improve full-cycle applicability. Two weeks after injection, the effectiveness of the system was visually evaluated by the albino phenotype caused by *GhCLA1* gene silencing, and the expression of the target gene (such as *GhERF49*) was detected by qRT-PCR to confirm the silencing efficiency.

### 4.4. Inoculation Treatment and Disease Index Statistics

After 2 weeks of growth of cotton with expression of gene *GhERF49* silenced by VIGS technology, *Verticillium dahliae* was inoculated using the root irrigation method. The culture conditions were day temperature 28 °C/night temperature 22 °C, light cycle 12 h light/12 h dark. The 2-week-old *Arabidopsis thaliana* was treated using the root dipping method. Each plant in the treatment group was dipped in conidial suspension for 5 min, and each plant in the control group was dipped in Czapek–Dox liquid medium for 5 min. All plants were then transplanted into fresh soil. The culture conditions were day temperature 28 °C/night temperature 22 °C, light cycle 12 h light/12 h dark. About 2 weeks after inoculation, the disease grade was recorded and the disease index was calculated according to the grading standard of cotton and *Arabidopsis* Verticillium wilt at the seedling stage. Disease was classified into five grades [24].

### 4.5. Design Primers

Primer Premier 5 was used to design fluorescent quantitative RT-PCR primers. The detailed sequence of primers is shown in Table 1. Primers were synthesized by Beijing Meiji Sanger Biomedical Technology Co., Ltd, Beijing, China.

### 4.6. qRT-PCR Analysis

Leaves of *Arabidopsis thaliana* and cotton before and after inoculation with *Verticillium dahliae* were used as materials for leaf RNA extraction and reverse transcription into cDNA. The lignin-related genes of *Arabidopsis thaliana* and cotton were used as detection objects, and expression changes in lignin-related genes were detected by qRT-PCR. qRT-PCR analysis was performed on *PAL4*, *C4H*, *F5H*, *CAD* and *CCoAOMT1* in the lignin biosynthetic pathway to explore the relationship between *AtERF49* and its homologous gene *GhERF49* with the lignin biosynthetic pathway. *Arabidopsis AtUBC30* was used as an internal reference gene, and cotton *GhHISTONE3* was used as a reference gene.

### 4.7. Fungal Recovery and Observation of Cotton Plant Cutting

At 14 days post-inoculation (dpi) with *Verticillium dahliae*, the same stem parts of TRV:*GhERF49* and TRV:00 plants with silenced *GhERF49* expression were sampled and placed on PDA medium containing cephalosporin at 25 °C under dark conditions in the incubator.

The cotyledon nodes of TRV:*GhERF49* and TRV:00 plants with silenced *GhERF49* expression were cut with a blade. The stems were dissected longitudinally, observed under a stereomicroscope, and photographed.

### 4.8. Trepan Blue Staining

The trepan blue working solution was prepared by diluting stock solution (containing 0.1% trepan blue) with 95% ethanol at a 1:2 (*v*/*v*) ratio, resulting in a final working concentration of approximately 0.033%. Leaves were placed in a microcentrifuge tube, immersed in the trepan blue staining solution, vacuum-infiltrated for 30 min, incubated at room temperature for 5 h, heated in a 90 °C water bath for 10 min, and cleared with a 2.5 g/mL chloral hydrate aqueous solution (replaced every 3–4 h) until the leaves became transparent.

## Figures and Tables

**Table 1:** Primers used for fluorescent quantitative PCR.

Primer Name	Primer Sequence (5′–3′)
GhC4H1-F	GATGCAAAGCTTGGTGGGTATGAC
GhC4H1-R	ACTTGTTAAATCAAAACACCCTTGGCTT
GhCCoAOMT1-F	AAGAAGGGCCTGCAATGCCAGTT
GhCCoAOMT1-R	GGTAACGGTGGTTCATTTGAGGCGA
GhF5H1-F	CGACGGTAGCATAGAACATCC
GhF5H1-R	CAACAAGCAAGATCATTGACCT
GhCAD6-F	GCTTCCAGCAACATCCACGAC
GhCAD6-R	AGGATTGTTGATGACGCCTGAC
AtCCoAOMT1-F	GGGTTTACCGATCATTGAGA
AtCCoAOMT1-R	CACCAACAGGGAGCATACAG
AtPAL4-F	CGGCGCCGGGGACACGTC
AtPAL4-R	GCGGCTTCGATCTGACCG

## Data Availability

The data and materials that support the findings of this study are available from the corresponding authors upon reasonable request.

## Abbreviations

The following abbreviations are used in this manuscript:

cDNA	Complementary DNA
VIGS	Virus-induced gene silencing
